# High Prevalence of Bidirectional Intimate Partner Violence Among Women With Infertility Attending a Reproductive Center in Guangdong, China: A Cross‐Sectional Study

**DOI:** 10.1155/jonm/4024244

**Published:** 2026-11-15

**Authors:** Chun-fang Tang, Mao-ling Zhang, Wei-ning Zhou, Miao-hong Chen, Xiao-juan Lu, Mei-ling Xia, Ya-qing Wang, Qi-yuan Lyu

**Affiliations:** ^1^ Reproductive Health and Infertility Center Guangdong Women and Children Hospital Guangzhou China e3861.com; ^2^ Medical Genetics Center Guangdong Women and Children Hospital Guangzhou China e3861.com; ^3^ School of Nursing Jinan University Guangzhou China jnu.edu.cn

**Keywords:** associated factors, intimate partner violence, prevalence, women with infertility

## Abstract

**Background:**

Infertility is associated with considerable psychosocial stress and may increase vulnerability to intimate partner violence (IPV). However, limited evidence is available regarding IPV among women with infertility attending a tertiary reproductive center in Guangdong Province, China.

**Aim:**

This study aimed to investigate the prevalence and associated factors of IPV among women with infertility at a tertiary reproductive center in Guangdong Province, China.

**Methods:**

A cross‐sectional study was conducted among 343 women receiving infertility treatment at a tertiary reproductive center in Guangdong Province, China, between August 1, 2021, and July 31, 2022. Convenience sampling was used. Data were collected using a sociodemographic questionnaire, the Chinese version of the Conflict Tactics Scale‐Short Form (CTS2S), and the Social Support Rating Scale (SSRS). Logistic regression analysis was performed to identify factors associated with IPV.

**Results:**

The analysis showed that the prevalence of IPV in women with infertility was 43.7%, with 77 participants experiencing multiple forms of violence. The prevalence of women as victims of IPV was 35.3%, and the prevalence of women as perpetrators of IPV was 37.3%. The results of regression analysis showed that spouse’s age, social support, and duration of infertility were significantly associated with the experience of IPV in women with infertility (*p* < 0.05).

**Conclusion:**

IPV was highly prevalent among women with infertility attending a reproductive center in Guangdong Province and was associated with infertility duration and social support. Routine IPV screening, psychosocial assessment, and multidisciplinary support may help reproductive healthcare professionals identify and support women at risk.

**Implications for Nursing Management:**

Nursing managers should strengthen IPV‐related training, establish screening protocols within reproductive healthcare settings, and promote multidisciplinary collaboration. Holistic couple‐oriented assessment should be considered when screening for IPV. Psychosocial support and timely referral should be provided not only for women with infertility affected by IPV but also for male partners when violent relationship behaviors are identified. Notably, information about violence experienced by male partners was reported exclusively by female participants in this study, without direct assessment from male partners.

## 1. Introduction

So‐called infertility refers to the inability to achieve pregnancy without contraception, despite regular sexual intercourse (at least twice a week) throughout the year [1]. According to World Health Organization (WHO) statistics, the global prevalence of infertility is 17.5%, and in some developing countries, it is as high as 30% [2]. In China, the number of patients attending infertility clinics is increasing by hundreds of thousands every year, reaching as high as 15.5%–25% [3].

The WHO defines intimate partner violence (IPV) as physical, sexual, and psychological violence perpetrated by a current or former intimate partner (including spouse, boyfriend/girlfriend, dating partner, or long‐term partner). The main manifestations are physical aggression, intentional violence against the partner, including kicking, pushing, and shoving; psychological abuse, coercion, threats, humiliation of the victim; and various forms of sexual coercion or forcing the victim to have sex with him [4]. IPV experienced by women with infertility is not only a serious violation of victims’ human rights but also hurts health. In addition to physical injuries such as headaches, bruises, and fractures, victims are prone to mental health problems such as depression and anxiety [5].

The United Nations (UN) Sustainable Development Agenda 2030 sets out the development goal of eliminating violence against women [6]. In the past period, there has been a deeper understanding of IPV, but we also need to recognize that some countries, including China, still consider this type of violence to be an internal problem within the family [7]. Most studies have focused on migrant women or pregnant women, and there are very few studies on women with infertility in China [8, 9].

Previous studies from some countries have found that the associated factors of IPV experienced by women with infertility are mostly sociodemographic factors. Turkey and Nigeria have conducted research on IPV experienced by women with infertility and focused on the associated factors [5, 10]. Iliyasu et al. believed that participants’ education level and occupation, and spouse’s education and occupation, are important associated factors of IPV [10], and Aduloju et al. believed that participants’ education and occupation are not associated factors of IPV [11]. The results of researchers in different countries are not the same, and the domestic analysis of IPV experienced by women with infertility in China is inadequate. At present, few people in China pay attention to IPV in infertility. IPV experienced by women with infertility often goes unreported due to stigma and social traditions. IPV against women with infertility is still at an exploratory stage in China today.

Despite growing international attention to IPV among women with infertility, evidence from China remains limited, particularly within reproductive healthcare settings. Furthermore, previous studies have primarily focused on prevalence estimates, while the implications for nursing management and reproductive healthcare practice have received insufficient attention. Considering the strong sociocultural expectations surrounding childbearing in China, women with infertility may experience unique psychosocial vulnerability and relationship stress. Therefore, this study aimed to investigate the prevalence and associated factors of IPV among women with infertility in Guangdong Province and to provide evidence for nursing management, early screening, and psychosocial intervention strategies in reproductive healthcare settings.

## 2. Methods

### 2.1. Study Design

This study was conducted in a cross‐sectional and descriptive design.

### 2.2. Participants and Procedure

This study was conducted and reported in accordance with the Strengthening the Reporting of Observational Studies in Epidemiology (STROBE) guidelines. The sample size was calculated using the single‐population proportion formula for cross‐sectional studies:

(1) n=Z12−α/2p1−pd2,

where *Z* = 1.96 (95% confidence level), *p* represents the expected prevalence of IPV (18%) based on previous studies of women with infertility in China [1], and *d* = 0.05. The minimum required sample size was 227 participants. After accounting for an anticipated 20% nonresponse rate, the required sample size increased to 284 participants. A total of 343 participants were ultimately included in the study. Data were collected between August 1, 2021, and July 31, 2022. A consecutive convenience sample of 360 female infertility patients was taken for the study, of whom 17 refused to answer some of the questions in the questionnaire. A total of 343 valid questionnaires were obtained. Inclusion criteria were as follows: Patients diagnosed with female infertility after completion of various outpatient examinations and who were able to give informed consent with a primary school education or higher. The exclusion criteria were as follows: (1) Patients with tumors, serious chronic diseases, and physical disease activity disorders. (2) Patients with incomplete information or incomplete clinical data. Researchers first explained the study and questionnaire to the participants; those who gave consent to participate were given the self‐reported questionnaires. Participants were not accompanied by a male partner (their spouses) during the study.

### 2.3. Measurement

Our study was conducted using paper questionnaires collected in the field. The questionnaires consisted of three sections: a sociodemographic questionnaire, the Chinese version of the conflict strategies scale, and a Social Support Rating Scale (SSRS).

#### 2.3.1. Sociodemographic Questionnaire Personal Information Form

The personal information form was created by the researchers in line with the literature. The content includes age, residence, occupation, income, family expenses, whether there are smoking and drinking, no adverse pregnancy history, and spouse and wife infertility years.

#### 2.3.2. Chinese Version of the Conflict Strategies Scale

Conflict Tactics Scale‐Short Form (CTS2S) is a simplified version of 20 items drawn by Murray Straus in 2004 from the 78 items in the Revised Conflict Tactics Scales. CTS2S consists of 5 subscales, including injury, sexual violence, physical violence, psychological violence, and negotiation. It is now a widely used tool for measuring IPV [12]. It has been used in many countries [12]. In this study, the Chinese‐translated version of the Conflict Strategies Scale was used to investigate the prevalence of violence among women with infertility [8]. In addition, the Cronbach’s alpha coefficient of each subscale ranges from 0.72 to 0.93 [8]. In the current study, the Cronbach’s *α* coefficient of the CTS2S was 0.77. The Chinese version of the Conflict Strategies Scale asks participants about the number of violent situations in the past year (0 times, 1 time, and more); 0 times is recorded as 0, 1 time and more is recorded as 1, and the experience of different types of violence in the past year can be calculated. For this study, respondents were considered to be victims (women’s IPV by spouse) if they scored greater than 0 on Questions 4, 6, 10, 12, 14, 16, 18, and 20. Respondents were considered to be perpetrators (spouse’s IPV by women) if they scored more than zero on Questions 3, 5, 9, 11, 13, 15, 17, and 19. The Conflict Strategies Scale can be used to calculate the prevalence of different types of violence in the past year.

#### 2.3.3. SSRS

It is abbreviated as SSRS and has been used to examine three dimensions: support utilization, subjective support, and objective support. It was created by a Chinese researcher in 1986 [13]. In addition, Cronbach’s alpha coefficient reached 0.82, which shows that this scale has good validity and reliability [13]. In the present study, the Cronbach’s *α* coefficient for the SSRS was 0.78. The scale contains three dimensions and 10 items: the use of social, subjective, and objective support. The sum of these is the total social support score. If the total score is relatively high, it means that the social support is relatively sufficient.

### 2.4. Ethics Approval

Ethical approval was obtained from the Institutional Review Board (IRB) of the study hospital (Approval No. 202101326). The study adhered to the tenets of the Declaration of Helsinki, as revised. All participants were assured that their data would be kept confidential, that participation was completely voluntary, and that they could withdraw from the study at any time. Informed consent was obtained from all participants before enrollment.

### 2.5. Data Analysis

Statistical analyses were performed using IBM SPSS Statistics Version 28.0 (IBM Corp.). Descriptive statistics were used to summarize participants’ sociodemographic and clinical characteristics. Continuous variables were presented as means and standard deviations, whereas categorical variables were expressed as frequencies and percentages.

The normality of continuous variables was assessed using Q–Q plots and P–P plots. Independent samples *t*‐tests and chi‐square tests were conducted for univariate analyses to compare differences between participants with and without IPV experience.

Variables with *p* < 0.05 in the univariate analyses were entered into the multivariate logistic regression model to identify factors associated with IPV. Multicollinearity among independent variables was assessed using variance inflation factors (VIFs). Odds ratios (ORs) and 95% confidence intervals (CIs) were calculated. A two‐tailed *p* value < 0.05 was considered statistically significant.

## 3. Results

### 3.1. Participants’ Characteristics

A total of 360 women were contacted in this study, although it should be noted that 17 participants refused to answer some of the questions in the questionnaire. 343 valid questionnaires were returned, giving a response rate of 95.3%. The average age of the women with infertility in the study was 32.80 years (SD: 5.09). The average age of the spouses was 34.66 years (SD: 5.38). Most women with infertility were in their first marriage (88.3%). The majority of participants had a high school education or higher. Family expenses were mainly borne by both spouses and the wife (62.4%). The years of infertility were less than 5 years for 67.9% and 6 years or more for 32.1%. In univariate analysis, statistically significant associations were found with the patient’s place of residence (*p* = 0.030), spouse’s education level (*p* = 0.049), duration of infertility (*p* = 0.038), and spouse’s age (*p* = 0.020) (see Table 1).

**TABLE 1:** Sociodemographic characteristics of participants and comparison of the IPV among women with infertility (*n* = 343).

Characteristics	Participants (*n* = 343)	Abused (*n* = 150)	Nonabused (*n* = 193)	Test and *p* value
*Place of residence*				*x* ^2^ = 4.712 *p = *0.030
Urban	176 (51.3)	67 (44.7)	109 (56.5)	
Rural	167 (48.7)	83 (51.3)	84 (43.5)	

*Profession of women*				*x* ^2^ = 10.760 *p = *0.056
Unemployed	61 (17.8)	25 (16.7)	36 (18.7)	
Worker	64 (18.7)	36 (24.0)	28 (14.5)	
Farmer	11 (3.2)	8 (5.3)	3 (1.6)	
Science, education, culture, and health	17 (5.0)	7 (4.7)	10 (5.2)	
Self‐employed	54 (15.7)	24 (16.0)	30 (15.5)	
Others	136 (39.7)	50 (33.3)	86 (44.6)	

*Profession of spouse*				*x* ^2^ = 3.819 *p = *0.576
Unemployed	20 (5.8)	8 (5.3)	12 (6.2)	
Worker	61 (17.8)	32 (21.4)	29 (15.0)	
Farmer	14 (4.1)	8 (5.3)	6 (3.1)	
Science, education, culture, and health	16 (4.7)	6 (4.0)	10 (5.2)	
Self‐employed	81 (23.6)	33 (22.0)	48 (24.9)	
Others	151 (44.0)	63 (42.0)	88 (45.6)	

*Education level of woman*				*x* ^2^ = 10.605 *p = *0.060
Elementary school	12 (3.5)	10 (6.7)	2 (1.0)	
Junior high school	86 (25.1)	39 (26.0)	47 (24.4)	
High school	92 (26.8)	42 (28.0)	50 (25.9)	
College	15 (4.4)	6 (4.0)	9 (4.7)	
Undergraduate	131 (38.2)	49 (32.6)	82 (42.5)	
Graduate	7 (2.0)	4 (2.7)	3 (1.5)	

*Education level of spouse*				*x* ^2^ = 11.109 *p = *0.049
Elementary school	11 (3.2)	9 (6.0)	2 (1.0)	
Junior high school	79 (23.0)	39 (26.0)	40 (20.7)	
High school	103 (30.0)	47 (31.3)	56 (29.0)	
College	8 (2.3)	3 (2.0)	5 (2.6)	
Undergraduate	128 (37.4)	48 (32.0)	80 (41.5)	
Graduate	14 (4.1)	4 (2.7)	10 (5.2)	

*Family financial stress*				*x* ^2^ = 1.303 *p = *0.254
Yes	187 (54.5)	87 (42.0)	100 (51.8)	
No	156 (45.5)	63 (58.0)	93 (48.2)	

*Household expense bearer*				*x* ^2^ = 3.306 *p = *0.191
Women	8 (3.2)	6 (4.0)	2 (1.0)	
Men	121 (35.4)	53 (35.3)	68 (35.2)	
Both	214 (62.4)	91 (60.7)	123 (63.7)	

*Family type*				*x* ^2^ = 0.274 *p = *0.600
Live with spouse	177 (51.6)	75 (50.0)	102 (52.8)	
Live with parents	166 (48.4)	75 (50.0)	91 (47.2)	

*Type of marriage*				*x* ^2^ = 0.030 *p = *0.863
First marriage	303 (88.3)	132 (88.0)	171 (88.6)	
Remarriage	40 (11.7)	18 (12.0)	22 (11.4)	

*Smoking habit of women*				*x* ^2^ = 0.133 *p = *0.715
Yes	3 (0.9)	1 (0.7)	2 (1.0)	
No	340 (99.1)	149 (99.3)	191 (99.0)	

*Drinking habit of women*				*x* ^2^ = 1.227 *p = *0.268
Yes	7 (2.0)	5 (3.3)	2 (1.0)	
No	336 (98.0)	145 (96.7)	191 (99.0)	

*Smoking habit of spouse*				*x* ^2^ = 3.722 *p = *0.054
Yes	129 (37.6)	65 (43.3)	64 (33.2)	
No	214 (62.4)	85 (56.7)	129 (66.8)	

*Drinking habit of spouse*				*x* ^2^ = 0.301 *p = *0.583
Yes	92 (26.8)	38 (25.3)	54 (28.0)	
No	251 (73.2)	112 (74.7)	139 (72.0)	

*Duration of infertility (years)*				*x* ^2^ = 4.303 *p = *0.038
5 or less	233 (67.9)	93 (62.0)	140 (72.5)	
6 and above	110 (32.1)	57 (38.0)	53 (27.5)	

*Adverse pregnancy history*				*x* ^2^ = 0.039 *p = *0.843
Yes	101 (29.4)	45 (30.0)	56 (29.0)	
No	242 (70.6)	105 (70.0)	137 (71.0)	

*Mean ± SD*				
Age of women (years)	32.80 ± 5.09	32.24 ± 4.60	33.24 ± 5.41	*t* = −1.845 *p* = 0.066
Age of spouse (years)	34.66 ± 5.38	33.91 ± 4.76	35.24 ± 5.76	*t* = −2.331 *p = *0.020

### 3.2. The Prevalence of IPV Among Women With Infertility

Of the 343 patients, 150 couples (43.7%) had experienced IPV, with the highest prevalence of psychological violence at 145 (42.3%), followed by physical violence at 57 (16.6%), injury at 38 (11.1%), and sexual violence with the lowest prevalence at 21 (6.1%). The prevalence rate of women as perpetrators of IPV was higher (37.3%) than the prevalence rate of spouses as perpetrators (35.3%) (see Table 2).

**TABLE 2:** Prevalence of types of intimate partner violence against the women with infertility in this study.

Type of violence	Perpetrator of IPV		
	Either one of the couple	By spouse	By women
Psychological violence	145 (42.3)	112 (32.7)	121 (35.3)
Physical violence	57 (16.6)	31 (9.0)	34 (9.9)
Injury	38 (11.1)	22 (6.4)	22 (6.4)
Sexual violence	21 (6.1)	12 (3.5)	9 (2.6)
Any violence	150 (43.7)	121 (35.3)	128 (37.3)

In this study, the most common form of IPV among women with infertility was individual psychological violence. 73 individuals were subjected to a single form of violence (72 suffered only psychological violence and 1 suffered only physical violence). 49 individuals had experienced two types of violence. 22 individuals had experienced three types of violence. 6 individuals had experienced all four types of violence (see Figure 1).

**FIGURE 1:**
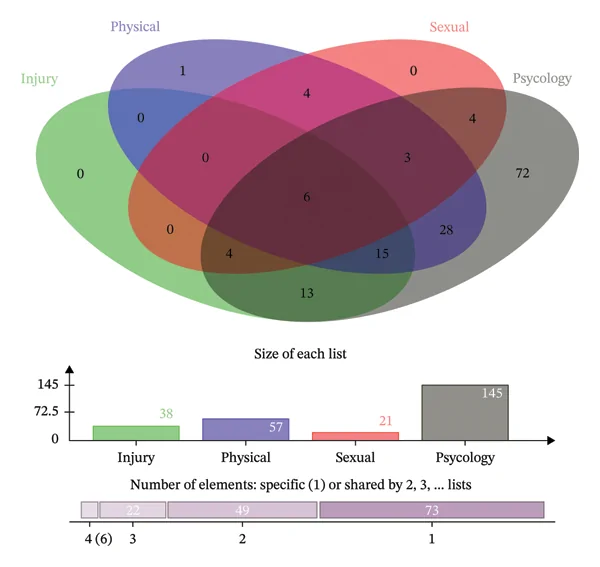
Frequency and overlap of types of IPV (abused = 150; nonabused = 193). The Venn diagram was generated using an online web tool. https://jvenn.toulouse.inrae.fr/app/example.html.

### 3.3. The Situation of Social Support in Women With Infertility

The study showed that participants with IPV experience had lower total social support scores (35.67 ± 7.56) than participants without IPV experience (39.65 ± 7.90). In independent samples *t*‐tests, objective support, subjective support, total social support, and support use were all lower and statistically significant for participants with IPV experience than for participants without IPV experience (*p* < 0.05) (see Table 3).

**TABLE 3:** Independent samples *t*‐test comparing SSRS scores between women with infertility with and without IPV experience.

Variables	Item number	Full score	Actual score$\;\left(\overset{¯}{\chi }\right.\pm$ S)			Test and *p* value
			Participants (*n* = 343)	Abused (*n* = 150)	Nonabused (*n* = 193)	
SSRS	10	12–64	37.91 ± 7.99	35.67 ± 7.56	39.65 ± 7.90	< 0.001
Subjective support	4	8–32	22.01 ± 5.15	20.81 ± 4.83	22.94 ± 5.20	< 0.001
Objective support	3	1–20	8.42 ± 3.10	7.71 ± 2.98	8.98 ± 3.08	< 0.001
Support utilization	3	3–12	7.49 ± 1.85	7.19 ± 1.80	7.73 ± 1.85	0.007

### 3.4. Logistic Regression Analysis of IPV Among Women With Infertility

In the logistic regression analysis, with the experience of IPV in women with infertility as the dependent variable, factors with a significance level of *p* < 0.05 in the univariate analysis were used as independent variables. The values of the dependent and independent variables are shown in Table 4. Of note, although spouse’s education level showed statistical significance in univariate analysis, it was no longer statistically significant after adjustment for other covariates in the multivariate logistic regression model and thus was not presented in Table 5. The results of regression analysis showed that spousal age, social support, and duration of infertility were significantly associated with the experience of IPV in women with infertility (*p* < 0.05), as shown in Table 5.

**TABLE 4:** The values for the dependent variable and independent variable.

Variables	Assignment
IPV	1 = *Abused*; 0 = *Nonabused*
Place of residence	1 = *Urban*; 2 = *Rural*
Education level of spouse	1 = *Elementary school*; 2 = *Junior high school*; 3 = *High school*; 4 = *College* 5 = *Undergraduate*; 6 = *Graduate*
Duration of infertility (years)	1 = 5 or less, 2 = 6, and above
Age of spouse (years)	Entering original data
SSRS	Entering original data

**TABLE 5:** Results of logistic regression analysis of factors analyzed by IPV experience.

Variables	B	SE	Wald *x* ^2^	*p*	OR	95%CI
Age of spouse (years)	−0.057	0.022	6.607	0.010	0.945	(0.905,0.987)
Duration of infertility (years)	0.570	0.247	5.351	0.021	1.769	(1.091,2.867)
Objective support	−0.096	0.041	5.509	0.019	0.908	(0.838,0.984)
Subjective support	−0.073	0.025	8.546	0.003	0.930	(0.886,0.976)

## 4. Discussion

### 4.1. Analysis of the Current Status of IPV Experience in Women With Infertility

IPV among women with infertility is a health problem. At present, China’s research in this field is still in the initial stage of development. Therefore, the current investigation of IPV and the analysis of associated factors in women with infertility are being studied to provide a more comprehensive clinical basis for the prevention of IPV in the future. This study showed that the prevalence of IPV was 43.7%. The results of this study were higher than those of Aduloju et al. and Iliyasu et al. in Nigeria [10, 11]. Several factors may explain this finding. (1) The study included records of violence from both spouses and not only from the infertile female spouse, which significantly increased the reporting rate. (2) The majority of subjects in this study had high school education, and the higher the level of education, the greater the ability of women with infertility to openly respond to and cope with violence [14]. (3) The Standing Committee of the 13th National People’s Congress of Guangdong Province formulated the implementation method of Guangdong Province (Guangdong Province Anti‐Domestic Violence Law) according to the characteristics of Guangdong Province. The subjects of this study were women with infertility in a tertiary hospital in Guangdong Province, and the survey was conducted after the conference; most of the study subjects had some knowledge and concern about IPV.

It should be noted that few published Chinese studies have reported IPV among women with infertility, with reported 12‐month IPV prevalence ranging from 10.5% to 26.9% in earlier domestic investigations [1]. Our observed overall IPV prevalence (43.7%) was comparatively higher. One plausible explanation is that our study measured bidirectional IPV, capturing violence perpetrated both by spouses and by women with infertility themselves, whereas most previous Chinese reports only focused on violence victimization experienced by women. Differences in study settings, measurement instruments, and participant characteristics may also contribute to such discrepancy.

The results of this study showed that the prevalence of IPV was lower than that of Sis Celik A [14]. This discrepancy may be explained by several factors. The reasons for this analysis could be (1) In Sis Celik A’s study, the subjects were infertile for more than 5 years, while in this study, most couples were infertile for less than 5 years. According to the analysis of related studies, the longer the duration of infertility, the greater the conflict between couples and the more likely the development of IPV [11]. (2) This is related to the different definitional scope of IPV. Sis Celik A’s study used the Infertility Violence Exposure Scale (IWEVDS), which includes five domains in the area of domestic violence: violence, punishment, social pressure, traditional practices, and exclusion. The definition of IPV experienced by women with infertility includes violence not only from the spouse, but also from the spouse’s brother, the spouse’s parents, and other family members, which may lead to a higher prevalence of violence than in this study.

The results show that the prevalence of psychological violence was the highest and the prevalence of sexual violence was the lowest. This is broadly consistent with the findings of a review study by Wang [1], which showed that psychological violence was the most common form of IPV among infertile patients. One possible explanation is the increasing prevalence of emotional neglect (“cold violence”) within intimate relationships in contemporary Chinese society. One study found that 35% of China’s 280 million families have varying degrees of “cold violence,” and in more than 60% of families where violence occurs, spouses ignore each other, resulting in psychological torture [15].

In contrast to most studies, the prevalence of IPV perpetrated by women with infertility in this study was higher than the IPV they experienced. (1) The reasons for this may be related to the increasing social status of women. Related studies have shown that social status is one of the factors influencing IPV [16], and in recent decades, the social status of women has increased greatly, making men with lower social status vulnerable to violence from their spouses when confronted with the fact of infertility. (2) Another reason may be related to the stress that women experience when confronted with the fact of infertility. Studies have suggested that stress occurs when there is a physical and/or psychological threat, which may lead to offensive or defensive aggression [17]. When women face the stress associated with infertility, it is possible that it may lead to violent behavior toward their spouses. Related studies have shown that mental health services play an important role in dealing with violence [18]. It is, therefore, necessary to train healthcare workers in appropriate psychological skills. Mental health services are provided to women with infertility as needed.

The types of violence very often occurred in an overlapping manner (see Figure 1), which is consistent with the findings of Silva ER’s study [19]. Related studies indicate the importance of early screening for IPV, which can help identify IPV victims, reduce abuse, and improve clinical and social outcomes for victims [20]. These findings support the integration of routine IPV screening into infertility care pathways. Early identification of IPV may facilitate timely referral, psychosocial support, and multidisciplinary intervention.

### 4.2. Analysis of Factors Influencing the Experience of IPV in Women With Infertility

In the present study, the spouse’s age was significantly associated with the experience of IPV among women with infertility. The older the spouse, the less likely women with infertility were to experience IPV, which is consistent with previous studies [21]. A study by Rayhan showed that younger spouses were more likely to perpetrate IPV compared to middle‐aged or older spouses [22]. On the one hand, as spouses grow older, they have a deeper understanding of family values, and their ability to self‐reflect and experience wisdom continues to accumulate, while they spend more time with and bond with their wives, which may reduce the prevalence of IPV [22]. On the other hand, as spouses age, they accumulate more wealth, which to varying degrees eases the economic pressure on infertile families and may indirectly reduce the prevalence of violence among women with infertility. It has been shown that as people age, their careers and incomes peak, and their financial decisions and confidence gradually increase [23].

Spouse’s education level exhibited a statistically significant association with IPV in univariate analysis; nevertheless, this association disappeared after adjusting for other confounding variables in multivariate logistic regression, suggesting that spouse’s education level was not an independent influencing factor for IPV among women with infertility. The crude association observed in univariate analysis might be confounded by other sociodemographic characteristics.

These findings suggest that younger couples undergoing infertility treatment may benefit from early psychosocial assessment and routine IPV screening. Reproductive nurses are well positioned to identify women at increased risk and coordinate timely psychological support and referral services. Psychological interventions and training will be provided for female infertility patients, and psychological clinics will be conducted to provide counseling and psychological consultation specifically for the psychological problems of infertile patients, to reduce the psychological stress among women with infertility, and for early screening of patients who have experienced violence. At the same time, healthcare providers can also extend medical services and psychological counseling to families by establishing multidisciplinary continuity of care, utilizing community health services and Internet remote online services, and regularly following up and tracking the experience of long‐term violence and psychological stress in female infertility patients to meet the diverse health needs of patients and families.

In this study, the longer the years of infertility, the greater the likelihood of suffering IPV. This is consistent with Aduloju et al.’s study [11]. Having children is a social responsibility of a family, and the inability to fulfill this reproductive responsibility can negatively affect the couple’s emotional state, marital relationship, plans, and self‐esteem. This situation can lead to marital conflict and violence.

Couples experiencing prolonged infertility may require additional psychosocial support throughout treatment. Reproductive healthcare teams should provide ongoing counseling, optimize communication regarding treatment progress, and facilitate access to multidisciplinary support services to alleviate psychological distress and potentially reduce the risk of IPV. At the same time, from the perspective of tertiary prevention, emphasis should be placed on identifying families in crisis of IPV at the level of medical institutions and communities. Finally, providing support to couples with infertility to improve patients’ perception of the fact of infertility and patiently explaining each stage of assisted reproduction and precautions to reduce patients’ psychological stress is beneficial to alleviate IPV [9].

This study found that social support was strongly associated with the development of IPV. This is consistent with the study of Ozturk et al. [24]. Social support has been confirmed by many studies as a protective factor or cachet factor for IPV [25]. Increasing social support can reduce individual dysphoria, reduce stress responses, and adopt positive coping styles in various ways, either directly or indirectly [26]. Social support includes family members, relatives, friends, group members with good traditional relationships, and various social networks. The so‐called objective support mainly refers to the actual or visible support; the subjective support refers to the emotional experience of individual understanding and support. In addition, support utilization refers to how individuals use the support they receive in social life [13].

Therefore, greater social support may serve as a protective factor against IPV. First, as medical professionals, they should provide care, help, and support to women with infertility in their daily medical work and provide patients with disease‐related information and materials promptly. Second, they should take the initiative to communicate with women with infertility to understand their social support, help them establish correct disease perceptions, mobilize the support system around them, and create a good social and medical environment for them. Self‐esteem is an important positive psychological resource that can reduce stress and improve mental health among women with infertility. In clinical practice, psychological interventions aimed at improving patients’ self‐esteem should be introduced. Finally, for female infertility patients who are closed and unwilling to use social support resources, medical professionals should encourage them to reduce their psychological burden; openly face the fact of infertility, fully utilize the help of family, relatives and friends, social groups, and related institutions to reduce the adverse effects of infertility; and positively accept treatment.

These findings highlight the importance of integrating routine IPV screening into infertility care. Nurses working in reproductive medicine settings are often the first healthcare professionals to establish continuous contact with women with infertility during diagnosis and treatment. Therefore, reproductive nurses may play a critical role in the early identification of IPV and psychosocial distress. Reproductive nurses should be trained to identify early signs of IPV and provide timely psychosocial support for women undergoing infertility treatment. Nurse managers should establish multidisciplinary referral pathways involving reproductive clinicians, psychologists, and social workers to improve support for women at risk of IPV.

Given the substantial prevalence of bidirectional IPV identified in this study, integrating IPV screening into routine infertility care may contribute to more comprehensive patient‐centered reproductive healthcare and improved psychosocial outcomes.

Because participants were recruited from a single tertiary reproductive center in Guangdong Province using convenience sampling, caution should be exercised when generalizing these findings to infertile populations in other regions of China.

## 5. Conclusion

These findings underscore the importance of integrating routine IPV screening and psychosocial assessment into infertility care. Given the bidirectional pattern of IPV observed among this sample, reproductive nurses should adopt a holistic couple‐oriented perspective during clinical assessment. Reproductive nurses and nurse managers may play a pivotal role in identifying couples at risk of reciprocal violent behaviors, delivering psychosocial support, and coordinating multidisciplinary referral for both women with infertility and their male partners when needed. It should be noted that data on violence experienced by male partners relied entirely on self‐reports from female participants; male partners were not directly surveyed in the current study. Future multicenter prospective studies that include direct assessment from both partners are needed to validate these findings across broader infertility populations.

## 6. Limitations

This study has several limitations. First, the cross‐sectional design precludes any inference of causal relationships between IPV and the associated factors identified. Second, participants were recruited from a single tertiary reproductive center in Guangdong Province using convenience sampling, which may have introduced selection bias and limited the representativeness and generalizability of the findings. Third, IPV information was self‐reported and therefore may have been subject to recall bias and social desirability bias. Finally, although the sample size exceeded the calculated minimum requirement, future multicenter studies with larger and more diverse populations are warranted to confirm and extend these findings.

## Author Contributions

All authors made a significant contribution to the work reported, whether that is in the conception, study design, execution, acquisition of data, analysis, and interpretation, or all these areas; took part in drafting, revising, or critically reviewing the article.

## Funding

No funding was received for this manuscript.

## Disclosure

All authors gave final approval of the version to be published; have agreed on the journal to which the article has been submitted; and agreed to be accountable for all aspects of the work.

## Conflicts of Interest

The authors declare no conflicts of interest.

## Data Availability

The data that support the findings of this study are available upon request from the corresponding authors. The data are not publicly available due to privacy or ethical restrictions.
